# Changes in characteristics of inpatient respiratory conditions from 2019 to 2021 (before and during the COVID-19 pandemic)

**DOI:** 10.3389/fpubh.2023.1268321

**Published:** 2023-11-09

**Authors:** Zahra Mojtahedi, Ji Yoo, Pearl Kim, Yonsu Kim, Jay J. Shen, Bing-Long Wang

**Affiliations:** ^1^Department of Healthcare Administration and Policy, School of Public Health, University of Nevada, Las Vegas, NV, United States; ^2^School of Medicine, University of Nevada, Las Vegas, NV, United States; ^3^School of Health Policy and Management, Chinese Academy of Medical Sciences & Peking Union Medical College, Beijing, China

**Keywords:** COVID-19, disparity, hospitalization, pandemic, respiratory

## Abstract

**Background:**

The COVID-19 pandemic has resulted in an increase in the number of individuals with respiratory conditions that require hospitalization, posing new challenges for the healthcare system. Recent respiratory condition studies have been focused on the COVID-19 period, with no comparison of respiratory conditions before and during the pandemic. This study aimed to examine hospital-setting respiratory conditions regarding potential changes in length of stay (LOS), mortality, and total charge, as well as socioeconomic disparities before and during the pandemic.

**Methods:**

The study employed a pooled cross-sectional design based on the State Inpatient Data Nevada for 2019 (prior to the COVID-19 pandemic) and 2020–2021 (during the pandemic) and investigated all respiratory conditions, identified by the International Classification of Disease, 10th Revision codes (*n* = 227,338). Descriptive analyses were carried out for the three years. Generalized linear regression models were used for multivariable analyses. Outcome measures were hospital LOS, mortality, and total charges.

**Results:**

A total of 227,338 hospitalizations with a respiratory condition were included. Hospitalizations with a respiratory condition increased from 65,896 in 2019 to 80,423 in 2020 and 81,018 in 2021. The average LOS also increased from 7.9 days in 2019 to 8.8 days in 2020 but decreased to 8.1 days in 2021; hospital mortality among patients with respiratory conditions increased from 7.7% in 2019 to 10.2% but decreased to 9.6% in 2021; and the total charges per discharge were $159,119, $162,151, and $161,733 from 2019 to 2021, respectively (after adjustment for the inflation rate). Hispanic, Asian, and other race patients with respiratory conditions were 1–3 times more likely than white patients to have higher mortality and LOS. Medicaid patients and non-White patients were predictors of a higher respiratory-related hospital total charge.

**Conclusion:**

Demographic and socioeconomic factors were significantly associated with respiratory-related hospital utilization in terms of LOS, mortality, and total charge.

## 1. Introduction

The COVID-19 pandemic had a significant impact on hospitals all across the world. As the virus spread, hospitals quickly became overwhelmed, especially with patients requiring respiratory assistance and acute care (1–3). The pandemic has also highlighted existing healthcare disparities, with marginalized communities disproportionately affected by COVID-19 and faced barriers to accessing healthcare resources (4–6). The spectrum of respiratory conditions in the hospital setting has been previously compared before and during the pandemic (7). Comparing respiratory conditions in hospital settings before and during the pandemic in terms of mortality, length of stay (LOS), total charges, and sociodemographic characteristics requires more clarification and provides valuable insights into the effectiveness of healthcare systems in managing the pandemic and its associated respiratory burden.

Before the pandemic, studies have shown that older age, male gender, lower socioeconomic status, race, and certain comorbidities were associated with higher rates of hospitalization, LOS, and increased mortality among patients with respiratory conditions (8–10). However, the sociodemographic factors associated with hospitalization, LOS, and mortality changed during the COVID-19 pandemic (11, 12). COVID-19 disproportionately affected marginalized communities, including people of color (12–14), which might be due to a variety of factors, such as limited access to healthcare, social determinants of health, being essential workers (non-remote work), higher-density living environments, and underlying medical conditions (12, 15, 16). Comparing data from before and during the pandemic will reveal how to enhance patient care and formulate policies for future pandemics or other crises.

Despite the apparent disproportionate impact of the COVID-19 pandemic on communities, this impact varied across areas and over time (17–21). Evidence indicates that there is a significant difference in terms of disparity across the United States (17, 18). For example, COVID-19-related death disparity was highest among Hispanics in California (19), while it was highest among African Americans in New York (20), implying that social determinants of health can be different even in the same country, and local public health prevention strategies might be needed for equitable resource allocation. Evidence also indicates that the distinction between the early effects of the pandemic and its late effects can also be important from a policy standpoint, and it should be taken into account in investigations focusing on the COVID-19 pandemic (6, 17). It has been previously shown that emergency department visits for patients with mental illnesses increased throughout the COVID-19 pandemic in Nevada; however, this increase was significantly greater in the early pandemic in 2020 than in the late pandemic in 2021, implying the necessity of more resource allocations for mental health early in a pandemic (6). Data from the CDC also indicate that during 2020–2021, age-adjusted mortality decreased for African American (−6.1%), Asian (−1.9%), and Hispanic people (−1.2%) but increased for non-Hispanic Native Hawaiian or other Pacific Islander (68.3%), multiracial (57.1%), White (35.1%), and non-Hispanic American Indian or Alaska Native populations (3.8%) (21), providing robust evidence that the early impacts of the COVID-19 pandemic on the healthcare system could be different from its late impact.

In the present study, each year of the pandemic was individually investigated in descriptive analyses in order to distinguish the early effects of the pandemic from its late effects. The study period is from 2019 to 2021 (one year before the pandemic and two years during the pandemic). The aims of this study were to (1) compare the volume of inpatient care with respiratory conditions before and during the pandemic; (2) compare LOS, mortality, and total hospital charges of inpatient care with respiratory conditions before and during the pandemic; and (3) examine potential socioeconomic disparities in LOS, mortality, and total hospital charges of inpatient care with respiratory conditions in Nevada.

## 2. Methods

### 2.1. Data

The State Inpatient Data Nevada (SIDN), which contains all hospitalizations of community hospitals in Nevada, was used to collect inpatient data for 2019 (one year before the pandemic) as well as two years during the pandemic (2020 and 2021) to investigate an immediate impact of the pandemic in 2020 and a non-immediate impact in 2021. The database encompasses information on all non-federal acute community hospitals in Nevada. All hospitalizations associated with respiratory conditions were identified using the International Classification of Diseases, 10th Revision (ICD-10) (22). These codes are listed in Supplementary Table S1. The University of Nevada, Las Vegas, institutional review board deemed this study exempt (IRB# UNLV-2023-10) because the database provides administrative data after complete de-identification. For the data analysis, a total of 227,338 hospitalizations with respiratory conditions (2019–2021) were included in this study.

### 2.2. Measures and data analysis

The present study investigated three dependent variables: hospital LOS, mortality, and charges associated with a respiratory condition one year before the pandemic and two years during the pandemic. Age, gender, race/ethnicity, and payer source had previously been linked to these dependent variables (23) and were thus included as independent variables in the regression model. In order to control for time and detect a potential trend, year was included in all regression analyses, as used by other prior studies (6).

Multiple visits from the same patient would be considered distinct hospitalizations because the data had been deidentified. To account for variations within hospitals due to the clustering effect, we utilized the generalized linear model for multivariable analysis and treated hospitals as random effects while estimating the fixed effect of the independent variables of individual hospital discharges (6). The binomial family with the link function of logit was used for mortality analysis. The identity link function was used in linear regression for LOS and total charges. All statistical analyses were conducted using SAS software version 9.4 (SAS Institute Inc.; Cary, NC, USA). All *p*-values > 0.05 (2-tailed) were considered statistically significant.

## 3. Results

Table 1 indicates the characteristics of hospitalizations with respiratory conditions from 2019 to 2021. The frequencies of hospitalizations with a respiratory condition among all hospitalizations were 65,896, 80,423, and 81,018 from 2019 to 2021, respectively. In all of these three years, more than 50% of the hospitalizations were made by men, and their proportions were 50.9, 53.6, and 52.6% from 2019 to 2021, respectively (value of *p* < 0.0001). Medicare was the most prevalent payer source, but its percentage was reduced during the pandemic while the percentage for private insurance increased (value of *p* < 0.0001). The proportion of White people who were hospitalized significantly decreased from 66.3% in 2019 to 59.3% in 2020 and 59.1% in 2021, whereas it increased for other racial/ethnic groups, particularly for Hispanic and Asian people (value of *p* < 0.0001). The percentage of in-hospital mortality was 7.7% in 2019 and peaked at 10.2% in 2020. These differences reached statistical significance (value of *p* < 0.0001). LOS and total hospital charges per discharge were also significantly higher in 2020 and 2021 compared to 2019 (value of *p* < 0.0001). LOS from 7.9 days in 2019 peaked at 8.8 days in 2020. Total charges per discharge from $159,119 in 2019 peaked at $162,651 in 2020 (Table 1).

**Table 1:** Characteristics of hospitalizations with a respiratory condition in Nevada (2019–2021 SIDN).

Characteristic	2019	2020	2021	Value of *p*
*N*	65,896	80,423	81,018	
Age, mean (St.d.), years	61.2 (22.0)	61.9 (19.7)	61.3 (20.3)	<0.0001
Gender				<0.0001
Female	49.1%	46.3%	47.45	
Male	50.9%	53.7%	52.6%	
Race/ethnicity				<0.0001
White	66.3%	59.3%	59.1%	
Hispanic/Latino	8.1%	11.1%	10.3%	
African American	12.3%	12.2%	12.9%	
Asians	5.1%	6.3%	6.6%	
Others	6.2%	8.8%	8.7%	
Payer Status				<0.0001
Private insurance	22.0%	26.2%	27.5%	
Medicare	54.3%	49.5%	47.7%	
Medicaid	18.5%	17.9%	17.8%	
Self-pay	2.5%	2.8%	2.6%	
No charge	0.4%	0.3%	0.2%	
Others	2.5%	3.3%	4.1%	
Mortality	7.7%	10.2%	9.7%	<0.0001
Length of Stays (St.d.), days	7.9 (12.9)	8.8 (13.2)	8.1 (10.6)	<0.0001
Total charges per discharge	$159,119	$162,651	$161,733	<0.0001

SIDN, State Inpatient Data Nevada; St.d., standard deviation.

Table 2 indicates factors associated with in-hospital mortality among hospitalizations with respiratory conditions in Nevada from 2019 to 2021. Moreover, compared to 2019, 2020 (OR = 1.31; CI = 1.26–1.37; value of *p* < 0.0001) and 2021 (OR = 1.25; CI = 1.20–1.31; value of *p* < 0.0001) were associated with higher odds of in-hospital mortality. Age and gender were positively and negatively, respectively, associated with in-hospital mortality during these three years (Table 2). Compared to the Whites, Hispanics (OR = 1.13; CI = 1.06–1.19; value of *p* < 0.0001) and Asians (OR = 1.21; CI = 1.31–1.29; value of *p* < 0.0001) with a respiratory condition had higher odds of in-hospital mortality during these three years. Compared to private insurance, self-pay (OR = 1.91; CI = 1.74–2.09; value of *p* < 0.0001) and no charge (OR = 1.53; CI = 1.14–2.07; value of *p* < 0.0001) were associated with higher odds of in-hospital mortality during these three years.

**Table 2:** Factors associated with mortality during hospitalizations with a respiratory condition in Nevada (2019–2021 SIDN^a^).

Independent variable	OR^b^	95% CI^c^	Value of *p*
Year*			
2019 (reference)			
2020	1.31	[1.26–1.37]	<0.0001
2021	1.25	[1.20–1.31]	<0.0001
Age*	1.40	[1.38–1.42]	<0.0001
Gender*			
Male (reference)			
Female	0.75	[0.72–0.77]	<0.0001
Race/Ethnicity*			
White (reference)			
African American	0.94	[0.90–0.99]	0.0399
Hispanic/Latino	1.13	[1.06–1.19]	<0.0001
Asian	1.21	[1.31–1.29]	<0.0001
Other races	1.28	[1.21–1.36]	<0.0001
Payer status*			
Private insurance (reference)			
Medicare	0.90	[0.86–0.94]	0.0112
Medicaid	1.03	[0.97–1.10]	0.3824
Self-pay	1.91	[1.74–2.09]	<0.0001
No charge	1.53	[1.14–2.07]	0.004
Others	1.27	[1.17–1.38]	<0.0001

^a^SIDN, State Inpatient Data Nevada. ^b^Odds ratio, ^c^Standard error. *p < 0.0001.

Table 3 indicates factors associated with LOS and total charges among hospitalizations with respiratory conditions in Nevada from 2019 to 2021 in a multivariable analysis. Compared to 2019, LOS in 2020 significantly increased. Hospital charges did not significantly increase either in 2020 or 2021 compared to 2019 (after controlling for the inflation rate). Age and gender were positively and negatively, respectively, associated with both LOS and total charges during these three years (Table 3). Compared to the White patients, Hispanic and Asian patients with a respiratory condition had both higher LOS and hospital charges during these three years. Medicare and self-pay patients were associated with lower LOS and total charges compared to those with private insurance, but Medicaid patients were associated with higher hospital charges and LOS during these three years.

**Table 3:** Factors associated with length of stay and total charges for hospitalizations with a respiratory condition in Nevada (2019–2021 SIDN^a^).

Independent variable	Parameter estimate	SE^b^	*p-*Value
Length of Stay			
Year*			
2019 (reference)			
2020	0.62893	0.064	<0.0001
2021	0.0641	−0.6	0.5495
Age*	0.65087	0.018	<0.0001
Gender*			
Male (reference)			
Female	−0.98544	0.051	<0.0001
Race/ethnicity*			
White (reference)			
Black	0.59159	0.081	<0.0001
Hispanic	1.01649	0.089	<0.0001
Asian	1.98345	0.109	<0.0001
Other races	3.32076	0.096	<0.0001
Payer status*			
Private insurance (reference)			
Medicare	−0.09295	0.068	0.1727
Medicaid	1.175	0.081	<0.0001
Self-pay	−1.81028	0.165	<0.0001
No charge	−0.88052	0.475	0.0637
Others	2.1665	0.149	<0.0001
Total charges			
Year*			
2019 (reference)			
2020	−2,095	1,336	0.1167
2021	−2,020	1,334	0.1299
Age*	12,497	367	<0.0001
Gender*			
Male (reference)			
Female	−27,726	1,067	<0.0001
Race/ethnicity*			
White (reference)			
Black	32,260	1,677	<0.0001
Hispanic	54,807	1,846	<0.0001
Asian	31,051	2,262	<0.0001
Other races	42,922	2,007	<0.0001
Payer status*			
Private insurance (reference)			
Medicare	−18,512	1,418	<0.0001
Medicaid	16,525	1,690	<0.0001
Self-pay	−48,662	3,429	<0.0001
No charge	−45,050	9,885	0.0012
Others	−27,026	3,092	<0.0001

^a^SIDN, State Inpatient Data Nevada. ^b^SE, standard error. *p < 0.0001.

## 4. Discussion

The present study investigated hospital utilization with a respiratory condition in Nevada from 2019 to 2021 (one year prior to and two years during the COVID-19 pandemic) in terms of hospital LOS, mortality, and total charges. The proportions of hospitalizations with respiratory conditions were also examined. While previous studies focused on respiratory-related hospitalizations during the pandemic (24), the current study compared respiratory-related hospitalizations before the pandemic (2019) and during the pandemic period (2020 and 2021) to investigate the pandemic’s disease and economic net burdens.

The results indicated a concerning trend in respiratory conditions in hospital settings during the COVID-19 period when compared to the pre-COVID-19 period. The frequency of hospitalization with respiratory conditions increased from 65,896 in 2019 to 81,018 in 2021 (Table 1), which may be attributed to the severe impact of COVID-19 pandemic on respiratory health or delayed healthcare seeking behavior due to the pandemic. The average LOS for patients with respiratory conditions also showed an increase. Prolonged hospital stays can put additional strain on healthcare resources (25). The reasons behind this longer LOS could be related to the complexity and severity of respiratory conditions during the pandemic, as well as potential challenges in providing timely and appropriate care (25). Moreover, it was found that the in-hospital mortality rate associated with a respiratory condition increased from 7.7% in the pre-COVID-19 period to 10.2 and 9.7% in 2020 and 2021, respectively (Table 1). The reasons for this increase could be multifactorial, including overwhelmed healthcare systems, resource constraints, and the increased severity of respiratory illnesses during the COVID-19 pandemic period (26). In addition to the increase in in-hospital mortality and LOS, the study highlighted a rise in total charges per discharge for patients with respiratory conditions. The increase in total charges during the COVID-19 pandemic period could be attributed to increased utilization of resources, higher demand for specialized care, longer LOS, and the additional expenses incurred due to infection prevention and control measures during the pandemic. It is worth mentioning that this increase did not reach statistical significance in the multivariable analysis (Table 3).

Current pandemic studies indicated that a higher proportion of hospitalizations due to COVID-19 were related to men, older adult, and racial/ethnic minority populations compared to their percentage in the population (17, 27, 28). Our study adds to the current literature indicating that during the pandemic period, compared to the pre-pandemic period, the proportion of males/females and non-Whites/Whites in hospitalized patients with a respiratory condition increased (Table 1). Moreover, our study identified certain demographic factors, including being older adult, male, and racial/ethnic minority groups, particularly Asians and Hispanics, that were associated with increased LOS, mortality and total charges for hospitalizations with respiratory conditions (Tables 2 3). These findings raise concerns about racial/ethnic disparities and the need for targeted interventions to address the specific needs of vulnerable populations during both the COVID-19 and non-COVID-19 periods. Another explanation for our findings could be that male and racial/ethnic minority groups might underutilize hospice and palliative care services, a type of care that lower hospital LOS, charges, and mortality. Previous studies have indicated that male and racial/ethnic minority groups are less proactive about choosing a hospital palliative care referral or enrolling in hospice when their medical care is futile, potentially leading to longer hospital LOS and higher hospital charges and mortality rates (23, 29, 30). This mechanism is also applied to the Medicaid recipients (29).

The findings of our study are consistent with previous studies showing that race and ethnicity are related to hospital outcomes (17, 30), but Asian populations were the most vulnerable race/ethnicity in Nevada in terms of hospitalization with respiratory conditions. Asian populations are heterogeneous. Nevada has a sizable native Hawaiian community (31), an Asian population that has been frequently reported to struggle with health disparities (32). In contrast to Nevada, Hispanics and African Americans were the most vulnerable populations during the COVID-19 pandemic in California and New York, respectively (19, 20). The variation in the association of certain races/ethnicities with outcomes based on geographic region suggests that race/ethnic-centered localized policies are needed to address potential health disparities, particularly during respiratory-related pandemics.

This study had limitations that should be acknowledged. The study’s reliance on retrospective data may introduce inherent biases and limitations in data quality, completeness, and accuracy. Additionally, the study focused solely on data from hospitals and did not account for healthcare services provided in other settings, such as outpatient care or home healthcare, which could lead to an underestimation of the true burden of respiratory conditions in the population. Moreover, the study highlighted disparities in outcomes based on race/ethnicity, gender, and age, but the underlying reasons for these disparities were not thoroughly investigated, warranting further research to better understand the contributing factors. Another limitation can be related to ICD codes, which are not error-free (23). These codes were used to identify respiratory conditions. However, due to our high sample size, these inaccuracies can only have a minor effect on how we interpret our results. Finally, data for 2022 were unavailable and were not included in the current analysis, which could have offered helpful information about respiratory conditions in the aftermath of the pandemic.

In conclusion, the findings of this study highlight the significant impact of the COVID-19 pandemic compared to pre-pandemic on hospital utilization for respiratory conditions. Demographic and socioeconomic factors were significantly associated with respiratory-related hospital utilization in terms of LOS, mortality, and total charge. These associations underscore the importance of implementing strategies to mitigate the burden on healthcare resources, improve patient outcomes, and address health disparities. Such strategies may include strengthening healthcare infrastructure, ensuring equitable access to care, and developing effective treatment protocols for respiratory conditions, particularly during times of crisis.

## Data availability statement

The datasets presented in this article are not readily available because of the data user agreement between the authors and the data provider. Requests to access the datasets should be directed to the corresponding authors.

## Ethics statement

The studies involving humans were approved by University of Nevada Las Vegas. The studies were conducted in accordance with the local legislation and institutional requirements. Written informed consent for participation was not required from the participants or the participants’ legal guardians/next of kin in accordance with the national legislation and institutional requirements.

## Author contributions

ZM: Writing – original draft, Writing – review & editing. JY: Writing – review & editing. PK: Writing – review & editing. YK: Writing – review & editing. JS: Conceptualization, Data curation, Formal analysis, Investigation, Methodology, Resources, Software, Supervision, Validation, Writing – review & editing. B-LW: Conceptualization, Data curation, Formal analysis, Investigation, Supervision, Writing – review & editing.
