# Supplementary material for: Changes in characteristics of inpatient respiratory conditions from 2019 to 2021 (before and during the COVID-19 pandemic)
Source: Front Public Health. 2023 Nov 9;11:1268321. doi: 10.3389/fpubh.2023.1268321 (PMC10665887; doi:10.3389/fpubh.2023.1268321)
Supplement: Supplementary file 1 [file Table_1.DOCX]

ICD-10-CM codes for COVID-19 and respiratory conditions

| Descriptions | ICD-10-CM codes |
| --- | --- |
| tested positive for COVID | U07.1 |
| **CONFIRMED** - The patient billing record contains the ICD-10 Diagnosis code B97.29 AND the patient was diagnosed with certain symptoms | B97.29 |
| **SUSPECTED** - The patient was exposed to the COVID virus (Diagnosis Code: 'Z20828') AND the patient has symptoms. | Z20828 |
| Acute upper respiratory infections | J00-J06 |
| Influenza and pneumonia | J09-J18 |
| Other acute lower respiratory infections | J20-J22 |
| Other diseases of upper respiratory tract | J30-J39 |
| Chronic lower respiratory diseases | J40-J47 |
| Lung diseases due to external agents | J60-J70 |
| Other respiratory diseases principally affecting the interstitium | J80-J84 |
| Suppurative and necrotic conditions of the lower respiratory tract | J85-J86 |
| Other diseases of the pleura | J90-J94 |
| Intraoperative and postprocedural complications and disorders of respiratory system, not elsewhere classified | J95-J95.89 |
| Other diseases of the respiratory system | J96-J99 |
